# Health Literacy in Early Primary Education: A Multimodal Critical Analysis of Greek Grade 1 Textbooks

**DOI:** 10.3390/healthcare14040426

**Published:** 2026-02-08

**Authors:** Pelagia Soultatou, Charalampos Economou, Pantelis Bagos

**Affiliations:** 1 Department of Basic and Clinical Sciences, Medical School, University of Nicosia, UNIC Athens, 16777 Elliniko, Greece; 2 Department of Sociology, School of Social Sciences, Panteion University, Syngrou Avenue, 17671 Athens, Greece; economou@panteion.gr; 3 Department of Computer Science and Biomedical Informatics, University of Thessaly, 35100 Lamia, Greece; pbagos@compgen.org

**Keywords:** health literacy, schools, curriculum, books, Greece

## Abstract

**Background**: Early childhood is a key period for the development of health literacy, and school textbooks play an important role in shaping early health-related understandings. **Objectives**: This study examines how health is represented in Grade 1 primary school textbooks in Greece and how children are positioned in relation to health within the curriculum. **Methods**: Multimodal critical discourse analysis was conducted on thirteen state-approved Grade 1 textbooks (n = 1.271 pages) published by the Ministry of Education and distributed free-of-charge to all public primary schools in Greece. The dataset covers seven subject areas: Language, English Language, Environmental Studies, Physical Education, Visual Arts, Music and Literature. Analysis was informed by Nutbeam’s typology of functional, interactive and critical health literacy. **Results**: Health-related content appeared across all subject areas but was unevenly framed. Language textbooks and workbooks emphasized prescriptive routines and functional health literacy. Environmental Studies and Literature offered more opportunities for reflective and relational engagement with health. Physical Education and Visual Arts supported well-being through activity and creativity but included limited explicit reflection. Across the curriculum, critical health literacy was minimally represented. **Conclusions**: Grade 1 textbooks in Greece promote basic health behaviors but provide limited support for the development of critical health literacy in early primary education.

## 1. Introduction

Health literacy in youth constitutes a fundamental contribution to the formation of young people’s values, norms, attitudes, and behaviors [1,2]. In this frame, schools are widely recognized as ideal settings to accomplish this purpose [3]. However, as highlighted in recent works, empirical research on children’s health literacy remains relatively limited [4] with particular gaps in studies examining how health literacy is constructed and promoted within formal educational settings [5]. Moreover, studies examining health literacy among young people have been disproportionately conducted in English-speaking countries, limiting the geographical and cultural scope of the existing evidence base. Recent evidence on health literacy has emerged from diverse global contexts. For instance, health literacy has been associated with youth mental health in Japan [6] or food literacy or high school students in Iran [7,8].

According to the World Health Organization (WHO), health literacy encompasses both cognitive and social skills that enable individuals to access, understand, and use information to maintain and promote their health [9]. One of the most prominent typologies of health literacy has been suggested by Nutbeam, including three categories: functional health literacy refers to fundamental reading, writing, and numeracy skills required to understand and act upon simple health information. Interactive health literacy involves more advanced cognitive and social skills that enable individuals to engage with health information, communicate effectively, and apply knowledge across different contexts; critical health literacy encompasses the capacity to critically analyze information, understand the social determinants of health and use this understanding to exert greater control over personal and collective health-related decisions, linking health literacy explicitly to empowerment and social action [10].

Although interest in critical health literacy has increased, the body of empirical research remains limited, with this limitation being especially evident in studies focusing on children and childhood settings [3,11,12]. Critical Health Literacy (CHL) has emerged as a significant response to the limitations of dominant health literacy models that emphasize individual behavior and biomedical knowledge while neglecting the broader social, political, and economic determinants of health [13]. The COVID-19 pandemic exposed the insufficiency of functional literacy in addressing misinformation, uncertainty, and structural inequality, underscoring the need for critical engagement and collective responsibility [14]. Within education, CHL remains marginal, as school curricula often prioritize lifestyle and risk while ignoring equity and justice. Embedding CHL in early education offers a means to develop students’ critical awareness and civic agency in navigating complex health challenges.

From a critical pedagogy perspective, educational materials such as textbooks are deeply political instruments that participate in the reproduction of dominant ideologies [15]. Rather than serving as neutral conveyors of knowledge, they function as sites where power is encoded and contested. The “hidden curriculum” is conceptualized as the implicit transmission of norms, values, and social expectations—particularly around bodies, behavior, and responsibility—that operate beneath the surface of formal instruction. It has also been argued that school knowledge is selectively constructed, reflecting the interests of dominant groups while marginalizing alternative or subjugated knowledge. Textbooks, in this view, embody “selective traditions” that naturalize visions of the world, shaping what is considered legitimate, appropriate, or normal for learners [16]. As such, they are key cultural texts through which broader social inequalities and normative assumptions are reproduced under the guise of commonsense education [12].

To contextualize this study, it should be noted that the Greek primary education system includes neither a compulsory health education subject at the primary level nor a dedicated health education textbook. Instead, health-related content is dispersed across multiple subjects [17]. Consequently, children’s exposure to health concepts is fragmented and contingent on how individual subjects frame health-related themes. This structural arrangement constrains opportunities for systematic, coherent, and critical engagement with health literacy from the early years of schooling.

Despite the recognized importance of early childhood for health literacy development, relatively few studies have examined how health is constructed in textbooks used in the earliest years of compulsory schooling, particularly in non-English-speaking contexts. This study aims to explore how health-related content is constructed in Grade 1 textbooks in Greece, focusing on how health is framed and how children are positioned in relation to health knowledge and responsibility, through the lens of health literacy and critical pedagogy.

The research questions that frame the purpose of this work are:

1. How is health constructed and represented in Grade 1 Greek textbooks?
2. What types of health literacy (functional, interactive, critical) are promoted through textual content?
3. How are children positioned as health subjects through discourses of responsibility, behavior, and morality?

## 2. Materials and Methods

This study aims to explore how health literacy is conveyed in the content of school texts aimed for Grade 1 in public primary education of Greece. More specifically, the Nutbeam’s typology is applied to identify the type of health messages conveyed in textbooks. 

To respond to the above goal, this study adopts the qualitative paradigm, employing multimodal critical discourse analysis (MCDA) to examine how the notion of health is constructed and represented in Grade 1 Greek primary school textbooks. MCDA conceptualizes meaning as produced through the interaction of multiple semiotic resources, including language, images, layout, color, and material design [18]. Texts such as school textbooks are understood as multimodal ensembles in which discourse is realized not only through what is said, but through how visual and material choices shape interpretation and naturalize worldviews. Semiotic choices—such as representation, salience, framing, and modality—are emphasized to reveal how ideologies are subtly embedded and normalized. MCDA moves beyond linguistic content and interrogates how images, tasks, and pedagogic design work together to position learners, legitimize specific forms of knowledge, and constrain or enable particular ways of understanding social phenomena such as health and well-being. 

The dataset comprised thirteen officially approved Grade 1 textbooks authorized by the Hellenic Ministry of Education for use in Greek public primary schools. These textbooks, totaling 1271 pages, have been distributed free of charge during the 2024–2025 school year. Although originally published approximately two decades ago, they have not undergone substantive revision since their initial release. All materials were accessed through the national digital portal (ebooks.edu.gr), ensuring consistency and standardization across the national curriculum (Appendix A). 

The dataset includes list of books for the stated year and includes across seven subject areas: (a) Language (i.e., Greek), (b) English Language, (c) Environmental Studies, (d) Visual arts, (e) Music Education, (f) Physical Education, (g) and Anthology of Literary Texts (i.e., Literature) summarized in Table 1.

The dataset comprises the complete set of Grade 1 textbooks mandated for use in Greek public primary schools, excluding Mathematics, which was excluded a priori due to the absence of health-related content.

The unit of analysis consisted of text excerpts and visual material (Appendix B and Appendix C). Analysis proceeded through four iterative phases:

1. All thirteen textbooks, comprising a total of 1271 pages as reported in Table 1, were scrutinized in their entirety. Analytic memos recorded preliminary impressions of how health was framed, typical activity demands, and recurring pedagogical moves.
2. Manual, line-by-line open coding identified any explicit or implicit reference to health, as a multidimensional construct, comprising physical, social, emotional, spiritual and environmental domains adapted to Nutbeam’s typology.
3. Codes were collapsed into cross-textual themes organized along two dimensions:
  (a) Health content domain (physical, emotional, social, environmental);
  (b) Pedagogical discourse (prescriptive/behaviorist; reflective/dialogic; expressive/affective; experiential/embodied).
4. The emergent themes were then mapped to Nutbeam’s typology of functional, interactive and critical health literacy to classify how textbooks invite basic skills, social/interpretive engagement, or critical appraisal.

All coding and analysis have been conducted manually; a full audit trail of memos and evolving code lists was maintained. All transcripts were inductively open coded manually to allow for close engagement with the data and iterative refinement of codes throughout the analysis.

As the analysis was conducted by a single researcher, traditional inter-coder reliability measures were not applicable. To ensure rigor, the researcher maintained a reflexive analytic journal to document interpretive decisions and address potential bias. Additionally, peer debriefing sessions were conducted with an external qualitative research advisor to review coding decisions and discuss emerging interpretations. The coding framework and themes were refined iteratively through these processes to enhance coherence and analytic transparency. The analysis was conducted by the first author, a sociologist with a doctoral degree in critical discourse analysis in health education. The researcher is a bilingual speaker of the Greek and English Language. Peer debriefing sessions were conducted at key stages of the analytic process, leading to iterative refinement of the coding framework and thematic categories. Throughout the analytic process, the researcher maintained a reflexive stance by documenting interpretive decisions and reflecting on how their disciplinary positioning and linguistic background may have shaped data interpretation.

To ensure accessibility for an international readership, verbatim excerpts are used in the original language but also reported in English translation by the first researcher who is bilingual; original Greek was consulted throughout analysis. Excerpts are brief, representative, and anchored to the theme and subject in which they appear.

To enhance trustworthiness in a single-coder MCDA, the following strategies were employed:

i. Reflexive memoing was used throughout the analytical process to document theoretical assumptions, analytical decisions, and alternative interpretations of multimodal configurations.
ii. Peer debriefing with an external qualitative advisor was undertaken to review code–theme coherence and to interrogate instances of potential interpretive overreach.
iii. Negative-case searching was systematically applied to challenge emerging patterns, for example by identifying open-ended or reflective pedagogic prompts within otherwise prescriptive textbook sequences.

All materials analyzed are publicly accessible state textbooks distributed via the Ministry’s open portal (Appendix A). The study involved no human participants and therefore did not require ethics board approval under institutional/national guidelines. Short translated textual excerpts are reproduced under fair use for scholarly critique; no high-resolution images are reproduced.

## 3. Results

Health-related content was identified across all thirteen Grade 1 textbooks analyzed; however, its distribution, modality, and pedagogical framing varied considerably by subject area. Overall, functional health literacy was most prevalent across the corpus, while interactive health literacy appeared selectively and critical health literacy was rare and marginal. Health was primarily constructed through routine-oriented and behavioral discourses, with children most often positioned as compliant and self-regulating subjects rather than as reflective or critical agents. The results are presented thematically, reflecting the final stage of a four-phase iterative coding process.

### 3.1. Distribution of Health Content Across Subject Areas

Health-related meanings were present across all subject areas, though with uneven density and emphasis. Language textbooks and workbooks contained the most frequent references to hygiene, posture, nutrition, and behavioral regulation, typically embedded in everyday classroom routines. Environmental Studies textbooks addressed a broader range of health domains—physical, social, emotional, and environmental—while Physical Education, Music, Visual Arts, and Literature contributed health meanings more indirectly, often through embodied activity, creative practice, or narrative form. Across the corpus, health was most often framed at the individual level, with limited attention to collective or structural determinants.

### 3.2. Functional Health Literacy: Routines, Discipline, and Bodily Regulation

Functional health literacy dominated the dataset and was particularly prominent in Language and Physical Education textbooks. Health was commonly presented as a set of correct routines and behaviors, including handwashing, posture, nutrition, sleep, and exercise, conveyed through imperatives, declarative statements, and definitional explanations. Visual material reinforced this framing through sequencing, binary contrasts (e.g., correct/incorrect), and authoritative imagery, such as teachers or idealized children modeling expected behaviors. In these representations, children were positioned as responsible but compliant subjects, expected to internalize and enact health norms rather than question them. Physical Education textbooks further supported functional health literacy by foregrounding biological and anatomical knowledge, framing health as bodily function and movement efficiency.

### 3.3. Interactive Health Literacy: Participation and Embodied Engagement

Interactive health literacy appeared less frequently and was concentrated mainly in Environmental Studies, Music, Visual Arts, and selected Physical Education activities. In these contexts, health was constructed through participatory and dialogic practices, including group discussions, cooperative tasks, movement, and creative production. Environmental Studies included prompts inviting children to reflect on caring for themselves, others, and their environment, supported visually through depictions of cooperation and discussion. Music and Visual Arts contributed interactive health literacy primarily through embodied and expressive modes, where learning occurred through rhythm, imitation, movement, and material manipulation. In these cases, children were positioned as active participants and co-creators, although opportunities for explicit reflection remained limited.

### 3.4. Marginal Presence of Critical Health Literacy

Across the corpus, critical health literacy was largely absent. Few instances encouraged children to question health norms, evaluate information, or consider broader social, cultural, or environmental determinants of health. Where such potential emerged—most notably in Environmental Studies and Visual Arts—it remained implicit and weakly scaffolded, often relying on teacher mediation rather than textbook design. Environmental issues such as waste management and water use were typically framed as individual behavioral responsibilities, constraining opportunities for critical engagement with structural or systemic factors.

### 3.5. Positioning of the Child as a Health Subject

Across textual and visual modes, children were most positioned as self-regulating, obedient, and morally responsible subjects. Health was constructed as a marker of being a “good” child—one who follows rules, controls the body, and behaves appropriately. This positioning was particularly evident in Language textbooks and accompanying visuals, where bodily discipline and calm social behavior were repeatedly normalized. Alternative positions—such as the child as reflective, dialogic, or critically aware—were rare and appeared only sporadically, primarily in Environmental Studies and Literature, without sustained pedagogical support.

Table 2 presents illustrative multimodal excerpts drawn from different subject areas in the original language of the data, encompassing both textual and visual material. Each excerpt is classified according to the relevant health domain (i.e., physical, social, emotional, or environmental) and coded using Nutbeam’s health literacy typology (i.e., functional, interactive, and critical), resulting in composite codes (e.g., Physical–Functional, Physical–Interactive, Physical–Critical).

## 4. Discussion

Foundational scholarship on health literacy has established the educational setting as a key site for health literacy development and has outlined pedagogical conditions under which functional, interactive, and critical health literacy may be fostered [3,19,20]. A considerable body of research has disproportionately focused on curricular aims, teaching strategies, or classroom-based interventions [1,5,21]. While this literature has made significant contributions, it has largely overlooked school textbooks as conveyors of health knowledge and has insufficiently engaged with insights from critical pedagogy scholarship [15,16].

This study examined how health-related content is constructed in Greek Grade 1 textbooks and how young learners are positioned as learners within early primary education. Drawing on a multimodal critical discourse analysis, the findings demonstrate a discursive predominance of functional health literacy, with health framed primarily through behavioral instructions, individual responsibility, and normative rules. In contrast, opportunities for interactive and critical health literacy were limited across subjects and modes.

### 4.1. Recurrent Discursive Patterns of Functional Health Literacy

Health-related content across Grade 1 textbooks is largely oriented towards the transmission of basic knowledge and the regulation of children’s behavior. Health is presented as a set of practices—such as hygiene routines, dietary habits, and safety behaviors—that children are expected to follow. This emphasis corresponds closely with the concept of functional health literacy, which focuses on acquiring basic information and skills necessary to function within health-related contexts.

This pattern aligns with international research demonstrating that early primary curricula and textbooks tend to privilege functional health literacy over more advanced forms. Comparative curriculum analyses indicate that while interactive and critical health literacy are frequently acknowledged in policy documents, they are rarely operationalized in learning materials, particularly in the early years of schooling [4]. In this sense, the Greek textbooks analyzed in this study reflect a broader global tendency rather than an isolated national case.

### 4.2. Limited Opportunities for Interactive and Critical Engagement

Although some instances of interactive health literacy were identified—such as prompts encouraging children to recognize emotions or discuss everyday health-related situations—these were sporadic and rarely extended beyond guided responses. As reported in the results, activities seldom invited children to interpret health information, negotiate meaning with peers, or reflect on alternative perspectives. Opportunities for critical engagement, including questioning health norms or examining social and environmental determinants of health, were largely absent. Previous research suggests that delaying the development of interactive and critical health literacy until later educational stages may be problematic, as health-related attitudes and behaviors formed in early childhood often persist into adulthood [5]. Studies with children and adolescents further indicate that young learners can engage with health concepts in reflective and interpretive ways when learning environments support such engagement [22]. This finding is also consistent with prior research on Greek pre-service teacher education, which has identified a marginal presence of health education in undergraduate curricula and limited incorporation of critical pedagogy [23]. In the Greek context, the implementation of school health education programs remains constrained by structural and institutional barriers—including insufficient teacher training, limited incentives and support for teachers, inadequate school infrastructure, and the fragmented, optional status of health education within the curriculum—underscoring the need for a coherent national strategy and stronger curricular integration to support the development of critical health literacy [24].

### 4.3. Positioning of Children as Passive Health Subjects

A central contribution of this study lies in its analysis of how children are discursively positioned within textbook content. Across subjects, children are primarily constructed as passive recipients of expert knowledge, expected to internalize and reproduce prescribed health behaviors. This positioning reinforces a view of health as an individual responsibility detached from broader social, cultural, or contextual factors. Similar findings have been reported in textbook analyses from other educational contexts, where health education content is characterized by normative messaging and limited scope for learner agency [25]. 

### 4.4. The Role of Multimodality in Reinforcing Health Discourses

By multimodal analysis, this study extends existing research that has predominantly focused on written text. In line with previous research, images, page design, and visual cues consistently reinforce textual messages, often depicting idealized health behaviors and compliant child figures [26]. While multimodal elements enhance accessibility and engagement for young readers, they may also contribute to the normalization of specific health practices without encouraging critical reflection. This finding resonates with school-based health literacy research emphasizing that meanings around health are produced multimodally and embedded within cultural representations [27]. When both textual and visual modes converge to present health as individualized and behavior-focused, the scope for alternative interpretations or critical engagement is further constrained.

### 4.5. Structural Constraints of Curriculum and Textbook Design

Importantly, the dominance of functional health literacy observed in this study should not be attributed to shortcomings on the part of teachers or learners. Rather, it reflects broader structural constraints embedded in curriculum frameworks and textbook design. Comparative studies indicate that even in systems where health literacy is explicitly prioritized, learning materials tend to favor functional and, to a lesser extent, interactive skills, with critical health literacy remaining marginal [28]. Research on school-based health literacy initiatives further highlights the challenges teachers face in extending beyond prescribed content, particularly in early primary education where curricular demands, time constraints, and age-related expectations shape pedagogical practices [27]. As authoritative curriculum artifacts, textbooks play a decisive role in delimiting how health is conceptualized and enacted in classroom contexts.

### 4.6. Implications for Health Literacy Development in Early Primary Education

Overall, the findings suggest that while Greek Grade 1 textbooks support the acquisition of basic health knowledge, they provide limited scaffolding for the progressive development of interactive and critical health literacy. International literature increasingly argues that fostering health literacy solely at a functional level may be insufficient for addressing complex contemporary health challenges [4,5]. Integrating age-appropriate opportunities for dialog, interpretation, and contextualization into early primary materials does not require abandoning functional health education. Rather, it involves broadening textbook content to include reflective questions, multiple perspectives, and representations of health as socially situated. Such an approach could better align early primary education with health literacy frameworks that emphasize empowerment, agency, and lifelong learning.

### 4.7. Future Research Directions

Future research could examine how teachers mediate textbook health content and how children interpret health meanings in classroom contexts. Comparative studies across grade levels or national settings may also clarify how opportunities for interactive and critical health literacy evolve throughout primary education. In addition, further research could explore children’s engagement with visual and digital health-related resources as multimodal materials become increasingly prominent.

### 4.8. Limitations of the Study

Several limitations should be acknowledged. This study has several limitations. First, the analysis focused on official Grade 1 textbooks and did not examine classroom enactment or children’s interpretations. While textbooks function as authoritative curriculum artifacts shaping learning opportunities, they do not capture how content is mediated by teachers or experienced by learners. Second, the study is context-specific, analyzing textbooks from one national education system and a single grade level. Although this limits direct generalizability, similar patterns reported in international curriculum and textbook research suggest broader relevance. Third, the qualitative, multimodal critical discourse analysis employed is interpretive by nature. To enhance rigor, multiple coding phases, peer debriefing, and negative case analysis were used; however, alternative interpretations remain possible. Fourth, the analysis was conducted by a single researcher and conventional inter-coder reliability measures were not applicable; however multiple strategies were applied to enhance analytical rigor. Finally, although visual and layout features were systematically analyzed, children’s responses to these multimodal elements were not examined. 

## 5. Conclusions

This study examined how health-related content is constructed in Greek Grade 1 textbooks and how young learners are positioned as learners within early primary education. Using a multimodal critical discourse analysis, the findings demonstrate a clear predominance of functional health literacy, with health largely framed through behavioral guidance, individual responsibility, and normative expectations. Opportunities for interactive and critical engagement with health concepts were comparatively limited. By focusing on textbooks as authoritative curriculum artifacts, the study highlights how early health-related meanings are shaped not only through written text but also through visual resources. The analysis shows that both textual and visual modes tend to reinforce compliant and individualized understandings of health, potentially constraining children’s agency and critical engagement. These findings contribute to international research on health literacy in schools by providing empirical insight into how early schooling experiences operationalize health literacy. They suggest that while functional health literacy plays a pivotal role in early childhood, a more balanced integration of interactive and critical dimensions may better support more comprehensive health literacy development. Addressing this imbalance may help align early primary education with contemporary health literacy frameworks that emphasize empowerment, participation and lifelong learning.

## Figures and Tables

**Table 1:** Set of data.

No	Book Title	Pages
1.	Language Grade 1—Student Book, Volume A	82
2.	Language Grade 1—Student Book, Volume B	86
3.	Language Grade 1—Workbook, Volume A	86
4.	Language Grade 1—Workbook, Volume B	74
5.	English Language—Grade 1—Student Book & Workbook	145
6.	Environmental Studies—Grade 1—Student Book	162
7.	Environmental Studies—Grade 1—Workbook	58
8.	Visual Arts—Grades 1 & 2—Student Book	80
9.	Visual Arts—Grades 1 & 2—Workbook	98
10.	Music—Grade 1—Student Book—Grade 1	58
11.	Music—Student Workbook—Grade 1	58
12.	Literature—Grades 1 & 2—Student Book	174
13.	Physical Education for Grades 1 & 2—Student Book	110

**Table 2:** Health-related multimodal excerpts across remaining Grade 1 textbooks.

Subject	Textbook	Page	Mode	Greek Excerpt/Visual Description	English Translation (Beneath)	Health Domain	Nutbeam Code
Physical Education	Φυσική Aγωγή A′ & B′ Δημοτικού—Bιβλίο Μαθητή	44	Text	«Το ανθρώπινο σώμα αναπτύσσεται με τα χρόνια και αποτελείται από μυς, οστά και άλλα όργανα. H συνεργασία των μυών με τα οστά μάς δίνει τη δυνατότητα να κινούμαστε.»	“The human body develops over the years and consists of muscles, bones, and other organs. The cooperation of muscles and bones enables us to move.”	Physical	**FP**
Physical Education	Φυσική Aγωγή A′ & B′ Δημοτικού—Bιβλίο Μαθητή	45	Text	«H κίνηση μας βοηθά να αναπτυχθούμε σωστά και να ζούμε καλύτερα.»	“Movement helps us develop properly and live better.”	Physical	**FP**
Physical Education	Φυσική Aγωγή A′ & B′ Δημοτικού—Bιβλίο Μαθητή	46	Text	«Γνωρίζεις ότι το σώμα σου έχει 206 οστά; … Τα οστά ενώνονται μεταξύ τους με αρθρώσεις και σχηματίζουν τον σκελετό.»	“Do you know that your body has 206 bones? … Bones connect to each other through joints and form the skeleton.”	Physical	**FP**
Physical Education	Φυσική Aγωγή A′ & B′ Δημοτικού—Bιβλίο Μαθητή	47	Text	«Το σχήμα αυτό, στο μάθημα της γυμναστικής, θα το λέμε σωματικό σχήμα.»	“This shape (of the body), in physical education class, we will call ‘body schema/body shape’.”	Physical	**FP**
Music	Μουσική A′ Δημοτικού—Τετράδιο Εργασιών	12–13	Text + layout	«Γύρω-γύρω όλοι… Χέρια-πόδια στη γραμμή, όλοι κάθονται στη γη…» (rhythmic, rule-like chant presented as a children’s movement/game text).	“All around, everyone… Hands and feet on the line, everyone sits on the ground…”	Physical (embodied movement) + Social (group coordination)	**IP** (interactive, because movement is organized as a coordinated group practice rather than only factual knowledge)
Music	Μουσική A′ Δημοτικού—Τετράδιο Εργασιών	7–11	Visual + tasks	Visual pedagogy: Pages are structured as large image panels + blank spaces for children to mark/color/draw in response to sound, rhythm, and bodily imitation (e.g., “listen carefully”/“imitate”). The layout foregrounds doing (embodied response) rather than explanation.	Translation of function: The visual design prompts children to *respond physically and performatively* (listening → acting), building skills through interaction with prompts and images.	Physical (sensorimotor)	**IP**
Literature (Anthology)	Aνθολόγιο Λογοτεχνικών Κειμένων A′ & B′ Δημοτικού (“Το δελφίνι”)	54	Text	«[H οδοντόβουρτσα] Μια οδοντόβουρτσα σκληρή… “Τα δόντια σάπισέ του τα ώστε να μη με θέλει!”»	“[The toothbrush] A hard toothbrush… ‘Rot his teeth so that he won’t want me!’”	Physical (oral health)	**FP** (hygiene/teeth appear as “health content”, but framed humorously via personification rather than explicit instruction)
Literature (Anthology)	Aνθολόγιο Λογοτεχνικών Κειμένων A′ & B′ Δημοτικού (“Το δελφίνι”)	14–15	Text + illustration	Narrative + discipline/food: Children who “didn’t know the lesson” are left “νηστικά το μεσημέρι”; mothers bring bread/food to children at school (owl/partridge story).	“Hungry at midday” functions as a disciplinary consequence; food care appears as parental responsibility and moral concern.	Physical (food) + Social (discipline/authority)	**FSO** (social norms/authority around care and compliance are foregrounded; health is indirectly implicated through food deprivation/care)

## Data Availability

No new empirical data involving human participants were generated in this study. The coding framework and the translated illustrative excerpts used to support the findings of this study are available in the Appendix A, Appendix B and Appendix C.

## Abbreviations

The following abbreviations are used in this manuscript:

FP/IP/CP	Physical (Functional/Interactive/Critical)
FSO/ISO/CSO	Social (Functional/Interactive/Critical)
FE/IE/CE	Emotional (Functional/Interactive/Critical)
FSP/ISP/CSP	Spiritual (Functional/Interactive/Critical)

## Appendix A

**Table A1:** Textbooks included in the corpus (Grade 1 Greek primary education).

No.	Subject	Textbook Title (English Translation)	Official Code/File	Publisher/Authority
C1	Greek Language	Γλώσσα A′ Δημοτικού—Bιβλίο Μαθητή, Τεύχος A (Greek Language Grade 1—Student Book, Vol. A)	10-0002-02	Institute of Educational Policy (IEP), Hellenic Ministry of Education
C2	Greek Language	Γλώσσα A′ Δημοτικού—Bιβλίο Μαθητή, Τεύχος B (Greek Language Grade 1—Student Book, Vol. B)	10-0004-02	IEP, Hellenic Ministry of Education
C3	Greek Language	Γλώσσα A′ Δημοτικού—Τετράδιο Εργασιών, Τεύχος A (Greek Language Grade 1—Workbook, Vol. A)	10-0003-02	IEP, Hellenic Ministry of Education
C4	Greek Language	Γλώσσα A′ Δημοτικού—Τετράδιο Εργασιών, Τεύχος B (Greek Language Grade 1—Workbook, Vol. B)	10-0005-02	IEP, Hellenic Ministry of Education
C5	Environmental Studies	Μελέτη Περιβάλλοντος A′ Δημοτικού—Bιβλίο Μαθητή (Environmental Studies Grade 1—Student Book)	10-0014-02	IEP, Hellenic Ministry of Education
C6	Environmental Studies	Μελέτη Περιβάλλοντος A′ Δημοτικού—Τετράδιο Εργασιών (Environmental Studies Grade 1—Workbook)	10-0015-02	IEP, Hellenic Ministry of Education
C7	Physical Education	Φυσική Aγωγή A′–B′ Δημοτικού—Bιβλίο Μαθητή (Physical Education Grades 1–2—Student Book)	10-0025-02	IEP, Hellenic Ministry of Education
C8	Visual Arts	Εικαστικά A′–B′ Δημοτικού—Bιβλίο Μαθητή (Visual Arts Grades 1–2—Student Book)	10-0231-01	IEP, Hellenic Ministry of Education
C9	Visual Arts	Εικαστικά A′–B′ Δημοτικού—Τετράδιο Εργασιών (Visual Arts Grades 1–2—Workbook)	10-0023-02	IEP, Hellenic Ministry of Education
C10	Music Education	Μουσική A′ Δημοτικού—Bιβλίο Μαθητή (Music Education Grade 1—Student Book)	10-0017-02	IEP, Hellenic Ministry of Education
C11	Music Education	Μουσική A′ Δημοτικού—Τετράδιο Εργασιών (Music Education Grade 1—Workbook)	10-0018-02	IEP, Hellenic Ministry of Education
C12	Literature	Aνθολόγιο Λογοτεχνικών Κειμένων A′–B′ Δημοτικού (Anthology of Literary Texts Grades 1–2)	10-0020-02	IEP, Hellenic Ministry of Education
C13	English Language	Aγγλικά A′ Δημοτικού—Bιβλίο Μαθητή (English Grade 1—Student Book)	10-0186-BM	IEP, Hellenic Ministry of Education

## Appendix B

**Table A2:** Illustrative textual excerpts related to health literacy from Grade 1 textbooks.

Excerpt ID	Subject	Book	Greek Excerpt	English Translation	Health Domain	Nutbeam HL Code	Discursive Function	Child Positioning
A1	Greek Language	Student Book	«Το πρωί ξυπνάω, πλένομαι και τρώω πρωινό.»	In the morning I wake up, wash myself, and eat breakfast.	Physical	**FP**	Establishes routine hygiene and nutrition	Responsible, self-regulating child
A2	Greek Language	Workbook	«Κάθομαι σωστά στο θρανίο και γράφω προσεκτικά.»	I sit properly at my desk and write carefully.	Physical	**FP**	Bodily discipline through imperative language	Compliant task-doer
A3	Greek Language	Student Book	«Όταν κοιμάμαι καλά, έχω ενέργεια για παιχνίδι.»	When I sleep well, I have energy to play.	Physical/Emotional	**FP/FE**	Causal link between rest and well-being	Self-monitoring child
A4	Greek Language	Workbook	«Παίζουμε χωρίς να χτυπάμε τους φίλους μας.»	We play without hurting our friends.	Social	**FSO**	Moral regulation of social behavior	Socially responsible peer
E1	Environmental Studies	Student Book	«Πλένω τα χέρια μου πριν φάω.»	I wash my hands before I eat.	Physical	**FP**	Hygiene framed as unquestioned norm	Obedient, health-compliant child
E2	Environmental Studies	Student Book	«Φροντίζουμε το σώμα μας και το περιβάλλον μας.»	We take care of our body and our environment.	Physical/Environmental	**IP**	Links personal health with surroundings	Participatory caretaker
E3	Environmental Studies	Workbook	«Τι μπορείς να κάνεις για να μένεις υγιής;»	What can you do to stay healthy?	Physical	**IP**	Open-ended prompt encouraging reflection	Reflective agent
E4	Environmental Studies	Student Book	«Δεν πετάμε σκουπίδια στο πάρκο.»	We do not throw rubbish in the park.	Environmental/Social	**FSO**	Behavioral norm linked to collective good	Responsible community member
E5	Environmental Studies	Workbook	«Συζητάμε πώς βοηθάμε ο ένας τον άλλον.»	We discuss how we help one another.	Social/Emotional	**ISO**	Dialogic framing of care	Cooperative co-participant
E6	Environmental Studies	Student Book	«Το καθαρό νερό είναι σημαντικό για όλους.»	Clean water is important for everyone.	Environmental	**IP → CP (emergent)**	Introduces shared resource framing	Emerging civic subject

## Appendix C

**Table A3:** Visual material related to health literacy in Greek Language textbooks (Grade 1).

Visual ID	Book	Description of Visual Material	Health Domain	Nutbeam HL Code	Salience & Framing (MCDA)	Child Positioning
GL-V1	Greek Language—Student Book (Vol. A)	Illustration of a child washing hands at a sink, placed next to short routine sentences	Physical	**FP**	Central image; everyday realism; hygiene depicted as normal and unproblematic	Responsible, self-regulating child
GL-V2	Greek Language—Student Book (Vol. A)	Sequence of images showing morning routine (waking up, washing, dressing, school bag)	Physical	**FP**	Linear sequencing guides interpretation as “correct order”	Disciplined body following routine
GL-V3	Greek Language—Student Book (Vol. B)	Children sitting upright at desks, smiling and attentive	Physical/Social	**FP/FSO**	Frontal angle; calm posture; classroom order foregrounded	Compliant learner, socially adjusted
GL-V4	Greek Language—Workbook (Vol. A)	Illustration of a child holding a pencil correctly	Physical	**FP**	Close framing of hand; bodily technique isolated	Body trained for school productivity
GL-V5	Greek Language—Workbook (Vol. A)	Image of children playing quietly together	Social	**FSO**	Soft colors; harmonious composition	Well-behaved social subject
GL-V6	Greek Language—Workbook (Vol. B)	Cartoon of a child slouching vs sitting correctly (contrastive pair)	Physical	**FP**	Binary visual opposition (wrong/right posture)	Self-correcting body
GL-V7	Greek Language—Student Book (Vol. B)	Smiling children eating together at a table	Physical/Social	**FP/FSO**	Communal framing; food as shared norm	Socialized eater
GL-V8	Greek Language—Student Book (Vol. B)	Teacher figure standing above seated children	Social	**FSO**	High angle; authority embodied visually	Child positioned as subordinate learner
GL-V9	Greek Language—Workbook (Vol. B)	Visual cue reminding children to “work carefully” (icon + child image)	Physical	**FP**	Iconic simplification; repetition across pages	Attentive, compliant task-doer
GL-V10	Greek Language—Student Book (Vol. A)	Illustrations accompanying poems using water, heart, light metaphors	Emotional	**FE**	Symbolic imagery; low modality realism	Feeling subject, affective self

**Table A4:** Visual material related to health literacy in Environmental Studies textbooks (Grade 1).

Visual ID	Book	Description of Visual Material	Health Domain	Nutbeam HL Code	Salience & Framing (MCDA)	Child Positioning
ES-V1	Environmental Studies—Student Book	Illustration of a doctor holding a clipboard titled “O κατάλογος της υγείας”, surrounded by food images	Physical	**FP**	Central placement; medical authority iconography; checklist genre	Compliant follower of expert-defined routines
ES-V2	Environmental Studies—Student Book	Photograph of fruit adjacent to hygiene/nutrition text	Physical	**FP**	High color saturation; photographic realism signals truth and normality	Child positioned as consumer of “healthy” foods
ES-V3	Environmental Studies—Student Book	Children washing hands before eating	Physical	**FP**	Sequential framing; instructional realism	Obedient, self-regulating body
ES-V4	Environmental Studies—Student Book	Children sleeping peacefully in clean bedrooms	Physical	**FP**	Soft colors; calm composition	Idealized healthy child
ES-V5	Environmental Studies—Workbook	Visual sequence guiding tooth brushing	Physical	**FP**	Step-by-step instructional imagery	Disciplined health practitioner
ES-V6	Environmental Studies—Workbook	Children playing together calmly in shared spaces	Social	**FSO**	Eye-level angle; harmony foregrounded	Morally regulated social subject
ES-V7	Environmental Studies—Student Book	Illustration of clean park vs polluted park	Environmental	**FSO**	Binary contrast (clean/dirty); moral visual coding	Child as rule-abiding environmental citizen
ES-V8	Environmental Studies—Student Book	Children throwing rubbish into bins	Environmental	**FSO**	Action frozen mid-task; behavioral modeling	Responsible community member
ES-V9	Environmental Studies—Workbook	Visual prompt for group discussion (children seated in circle)	Social/Emotional	**ISO**	Circular composition; equal gaze relations	Participatory co-learner
ES-V10	Environmental Studies—Student Book	Illustration of water sources (tap, river) used to introduce clean water	Environmental	**IP**	Simplified schematic visuals	Emerging civic subject
ES-V11	Environmental Studies—Student Book	Image of children helping one another	Social/Emotional	**ISO**	Close proximity; affective cues	Caring, cooperative peer
ES-V12	Environmental Studies—Workbook	Visual showing “correct vs incorrect” behavior (e.g., hygiene, littering)	Physical/Environmental	**FP/FSO**	Binary opposition; moral clarity	Self-correcting subject
